# Effects of Extracts from *Trifolium medium* L. and *Trifolium pratense* L. on Development of Estrogen Deficiency-Induced Osteoporosis in Rats

**DOI:** 10.1155/2012/921684

**Published:** 2012-11-29

**Authors:** Urszula Cegieła, Joanna Folwarczna, Maria Pytlik, Grażyna Zgórka

**Affiliations:** ^1^Department of Pharmacology, Medical University of Silesia, Katowice, Jagiellońska 4, 41-200 Sosnowiec, Poland; ^2^Department of Pharmacognosy with Medicinal Plant Unit, Medical University of Lublin, Chodźki 1, 20-093 Lublin, Poland

## Abstract

Some plant species belonging to *Trifolium* L. genus are a source of isoflavones considered to exert phytoestrogenic activities. The aim of the present study was to examine the effects of standardized extract obtained from aerial parts of *Trifolium medium* L., in comparison with the extract of *Trifolium pratense* L., on the development of estrogen deficiency-induced osteoporosis in rats. Both *Trifolium* extracts, at doses corresponding to 10 and 20 mg/kg of isoflavone aglycones daily, or estradiol (0.2 mg/kg daily), were administered orally to ovariectomized (OVX) rats for 4 weeks. Serum bone turnover markers, bone mass, mineralization, and mechanical properties were studied. In OVX control rats, mechanical properties of the tibial metaphysis and femoral neck were strongly worsened in comparison with sham-operated control rats, and those of femoral diaphysis were unaffected. Estradiol counteracted the worsening of the tibial strength and increases in bone turnover markers. Both extracts significantly increased the strength of the femoral diaphysis and calcium and phosphorus content in the bone mineral, but only *T. pratense* extract increased the strength of the tibial metaphysis. In conclusion, effects of both *Trifolium* extracts differed from those of estradiol. It is possible that other than isoflavone extract constituents contributed to their skeletal effects.

## 1. Introduction

Phytoestrogens are plant derived substances with estrogenic activity, which, binding to estrogen receptors, may exert agonistic, antagonistic, or partial agonistic/antagonistic effects [1]. They are sometimes considered natural selective estrogen receptor modulators (SERMs); however there is only a weak association between the action of phytoestrogens and SERMs [2]. Due to the side effects of hormonal replacement therapy, there is an interest in using phytoestrogens to alleviate the menopausal symptoms, including development of osteoporosis. It is generally accepted that estrogen deficiency contributes to development of osteoporosis in postmenopausal women. 

Red clover (*Trifolium pratense *L.) is a well-known source of phytoestrogenic isoflavones. It contains mainly formononetin and biochanin A, and much smaller amounts of daidzein and genistein, mostly in glycosidic form [3, 4].

Although some efficacy of red clover extracts in the preventing of vasomotor symptoms was demonstrated in a meta-analysis of 5 clinical trials [5], more recently both lack of effects [6, 7] and favorable effects [8] were reported. The data on the long-term effects on the cardiovascular, breast, endometrial, and urogenital health, as well as bone health, are much scarcer [9]. There are clinical reports both on lack of significant effects [10, 11], and some favorable effects [12, 13] of red clover extracts on the skeletal system. Nevertheless, beneficial effects of red clover extracts on bones have been demonstrated in the experimental settings [14–16].

Previous phytochemical studies done by Zgórka [17] revealed that dried, aerial parts of zigzag clover (*Trifolium medium *L.) might constitute quite new and rich source of isoflavone phytoestrogens (especially formononetin and biochanin A). Total content of aglycone forms of isoflavones (native and those released from glycosides by hydrolysis), calculated for this species, was about threefold higher than in red clover and comparable with amounts reported in soybeans. 

*T. medium* is a rhizomatous perennial plant belonging to the Fabaceae (Leguminosae) family. Its trivial name is zigzag clover because of the tendency for the ascending stems to grow in zigzagged (curved) manner. As a representative of the clover genus, *T. medium* distinguishes by characteristic spherical flower heads composed of reddish purple flowers. In general appearance, *T. medium* is similar to commonly known red clover; however it possesses longer stalk, darker flower heads, and longer, lanceolate-elliptic leaflets without the noticeable pale “V” mark, occurring in the latter species. *T. medium *grows wild throughout Eurasia, especially in sunny grasslands, roadsides, waste grounds, and forest margins, often with poor soil. It is listed as a fodder crop in the Mediterranean region; however it is much less common than red clover [18, 19].

The effects of *T. medium *isoflavones on the skeletal system have not been studied so far. The aim of the present study was to examine the effects of the standardized extract obtained from aerial parts of *T. medium*, in comparison with the extract of *T. pratense*, on development of estrogen deficiency-induced osteoporosis in ovariectomized rats. Moreover, ovariectomized rats receiving estradiol served as a positive control.

## 2. Methods

*Trifolium* extracts were used. The standardized plant extracts were obtained from dried aerial parts of *T. medium *L. and *T. pratense *L. by ultrasound-assisted extraction with 50% (v/v) ethanol, followed by lyophilization. The standardization procedure, comprising the quantitation of bioactive isoflavones (aglycone forms) in hydrolysed clover extracts, was performed using reversed-phase high-performance liquid chromatography (RP-HPLC) combined with photo-diode array detection (PDA). The mean isoflavone content, calculated as the sum of four aglycones: formononetin (F), biochanin A (B), genistein (G), and daidzein (D), was 7.2 and 2.8% of dry weight, for *T. medium *and *T. pratense *extracts, respectively. The percent concentrations of aglycones, in isoflavone fractions, were as followed: 46.3% (F), 41.8% (B), 11.2% (G), and 0.7% (D) for *T. medium*, and 49.0% (B), 44.9% (F), 5.3% (G), and 0.8% (D) for *T. pratense. *Both extracts were used at doses corresponding to 10 and 20 mg/kg of total isoflavone aglycones/day, p.o.

Other drugs used included estradiol hemihydrate (Estrofem, Novo Nordisk A/S, 0.2 mg of estradiol/kg/day, p.o.), ketamine—Bioketan (Vetoquinol Biowet) and xylazine—Xylapan (Vetoquinol Biowet).

The study was carried out with consent of the Local Ethics Commission in Lublin, on 3-month-old female Wistar rats obtained from the Center of Experimental Medicine, Medical University of Silesia. The initial body mass of rats was 210–240 g. The rats were fed a soy-free diet *ad libitum*. The animals were switched from the standard laboratory diet to the soy-free diet on the day before the beginning of the extract or estradiol administration.

Bilateral ovariectomy with the access to the ovaries from the dorsal side was performed in general anesthesia induced by intraperitoneal injections of ketamine with xylazine. During sham operation, the ovaries were exteriorized only. The operations took place 7-8 days before the start of the drug administration.

The rats were divided into the following groups (*n* = 9-10 per group):

- sham-operated control rats,
- ovariectomized (OVX) control rats,
- ovariectomized rats receiving *T. medium* extract (10 mg isoflavone aglycones /kg p.o. daily),
- ovariectomized rats receiving *T. medium* extract (20 mg isoflavone aglycones /kg p.o. daily),
- ovariectomized rats receiving *T. pratense* extract (10 mg isoflavone aglycones /kg p.o. daily),
- ovariectomized rats receiving *T. pratense* extract (20 mg isoflavone aglycones/kg p.o. daily),
- ovariectomized rats receiving estradiol (0.2 mg/kg p.o. daily).

Estradiol or the extracts were administered by a gastric tube (p.o.) for 28 days, at a volume of 2 mL/kg. Control rats were administered the vehicle - tap water at a volume of 2 mL/kg p.o. Moreover, to mark the calcification front, the animals were given tetracycline hydrochloride (20 mg/kg, i.p.) one day before the start of drug or vehicle administration, and calcein (10 mg/kg, i.p.), after 3 weeks. 

The next day after the last drug administration, after 24-h fasting, the animals were killed by cardiac exsanguination, in full ketamine-xylazine anesthesia. From the sacrificed animals, estrogen-dependent organs (the uterus and thymus) and bones, the left and right femur, left and right tibia, and L-4 vertebra, were isolated. Immediately after isolation, the organs were weighed (with 0.1 mg accuracy). The length and diameter of left bones were measured with a caliper (with 0.01 mm accuracy). The bones were wrapped in gauze soaked in 0.9% NaCl solution and kept below −20°C until the mechanical tests were performed on thawed bones [20].

### 2.1. Bone Mechanical Properties Studies

Mechanical properties of the left femoral diaphysis, left tibial metaphysis, and the neck of the right femur were assessed using Instron 3342 500N apparatus with Bluehill 2 version 2.14 software. Mechanical properties of the left femoral diaphysis and left tibial metaphysis were studied using bending tests with three-point loading, as previously described [20–22]. The load was applied perpendicularly to the long axis of the femur in the midlength of the bone (distance between the supporting points: 16 mm) or to the proximal tibial metaphysis. The displacement rate was 0.01 mm/s. The load-displacement curves, obtained for each bone, representing the relationships between load applied to the bone and displacement in response to the load, were analyzed. Maximum load and displacement, energy, and stress for the maximum load, as well as fracture load and displacement, energy, and stress for the fracture load were assessed. Young's modulus was also determined. To determine moment of inertia in the cross-section, necessary for the calculations of the intrinsic bone mechanical parameters, it was assumed that the femoral diaphysis was an elliptical pipe, and the tibial metaphysis was a circular beam. For the femoral diaphysis, the transverse cross-sections of the right diaphysis were made in the midlength, and the inside and outside diameters were measured, according to [23], using Osteomeasure software (Osteometrics) for histomorphometric measurements. The mean diameter of the tibial metaphysis was measured using a caliper.

Mechanical properties of the femoral neck were studied using a compression test [22, 24]. The load was applied to the head of the femur along the long axis of the femur (displacement rate of 0.01 mm/s) and the maximum load (load causing the fracture of the femoral neck) was determined.

### 2.2. Bone Histomorphometric Studies

The right femurs were used to prepare histological specimens, as previously described [22, 25, 26]. Histomorphometric measurements were made using an Optiphot-2 microscope (Nikon), connected through an RGB camera (Cohu) to a computer, using Lucia G 4.51 software (Laboratory Imaging), with final magnifications of 200 and 500 times, or using Osteomeasure software (magnification 70 times).

The width of trabeculae in the distal epiphysis and metaphysis was measured in the longitudinal preparation from the femur. The area of the transverse cross-section of the cortical bone and the area of the transverse cross-section of the marrow cavity were determined in transverse cross-sections made from the femoral diaphysis in midlength of the femur. The periosteal and endosteal transverse growth of the femur was also measured.

### 2.3. Bone Mineralization Studies

To determine the mass of bone mineral (ash), the bones were mineralized at the temperature of 640°C for 48 h in the muffle furnace and weighed. The ratio of the mass of bone mineral to the bone mass was also determined as a substitute for bone mineral density measurements. 

Calcium and phosphorus content in the mineralized bones (dissolved in 6 M HCl and then diluted in distilled water) was determined colorimetrically, using a calcium reagent set and a phosphorus reagent set, both produced by Pointe Scientific. 

### 2.4. Biochemical Studies

Serum estradiol concentrations were studied by an ELISA method (Mouse/Rat Estradiol ELISA, Calbiotech, Inc.). Serum osteocalcin levels were determined using an enzyme immunoassay (Rat-MID Osteocalcin EIA, Immunodiagnostic Systems Ltd.). Serum levels of type I collagen fragments released from bone during bone resorption were determined by an enzyme immunoassay (RatLaps EIA, Immunodiagnostic Systems Ltd.). Moreover, serum concentrations of calcium, phosphorus, and total cholesterol were assayed colorimetrically, using Pointe Scientific reagent sets.

### 2.5. Statistical Analysis

The results are presented as arithmetical means ± SEM. Statistical estimation was carried out based on the analysis of variance. After confirmation of statistically significant differences in one-way ANOVA (*P* < 0.05), further analysis was carried out by means of Duncan's *post hoc *test. In case of a lack of normality (Shapiro-Wilk's test) or of homogeneity of variance (Levene's test), nonparametric tests were used: Kruskal-Wallis ANOVA and Mann-Whitney *U* test. The results obtained in each experimental group were compared with those of the sham-operated control rats and ovariectomized control rats.

## 3. Results

### 3.1. Body Mass Gain, Mass of Estrogen-Dependent Organs, and Serum Levels of Estradiol and Total Cholesterol

The ovariectomized control rats had significantly decreased serum estradiol level and the uterus mass. The mass of thymus, as well as the body mass gain and serum cholesterol level, were significantly increased (Table 1). Neither of the investigated *Trifolium* extracts significantly affected the abovementioned estradiol-dependent parameters. The changes in the uterus mass, body mass gain and serum cholesterol level were partially counteracted by administration of estradiol (0.2 mg/kg daily). 

There were no effects of the treatments on serum calcium and phosphorus levels (not shown).

### 3.2. Bone Mass and Mineralization

Estrogen deficiency in the ovariectomized control rats caused significant decreases in the mass of the tibia, femur, and L-4 vertebra, expressed as the ratio to the body mass, in comparison with the sham-operated rats (Table 2). The ratio of the mass of bone mineral to the bone mass was significantly decreased in the long bones. There was no effect of estrogen deficiency on the calcium and phosphorus content in the bone mineral.

Extracts of *Trifolium *species did not affect the bone mass expressed as the ratio to the body mass nor the content of bone mineral in the bone (the ratio of mass of bone mineral to bone mass) in comparison with the ovariectomized control rats. Both *T. medium* and *T. pratense* extracts at two isoflavone doses significantly increased the content of calcium and phosphorus in the bone mineral of the tibia and L-4 vertebra, but not of the femur. 

Administration of estradiol to the ovariectomized rats did not significantly affect the ratio of bone mass to body mass, nor the ratio of the mass of bone mineral to the bone mass. Administration of estradiol also did not significantly affect the content of calcium and phosphorus in the bone mineral.

### 3.3. Mechanical Properties of Cancellous Bone (Tibial Metaphysis)

Estrogen deficiency very strongly worsened mechanical properties of the cancellous bone (Table 3). In the ovariectomized control rats, the maximum load and energy accumulated for the maximum load, as well as maximum stress and Young's modulus, were significantly decreased in relation to the sham-operated control rats. The mechanical parameters for the fracture point also significantly decreased (not shown).

The extract of *T. medium* in both doses did not affect the mechanical properties of the tibial metaphysis, whereas extract of *T. pratense* significantly increased the maximum load sustained by the bone in the ovariectomized rats. However, energy for maximum load and the intrinsic mechanical parameters (maximum stress and Young's modulus) remained unchanged.

The supplementation of estradiol significantly increased both the maximum load and maximum stress. Although the accumulated energy for the maximum load remained unchanged, Young's modulus did not significantly differ from the sham-operated control rats. 

### 3.4. Mechanical Properties of Cortical Bone (Femoral Diaphysis)

**Figure 1:**
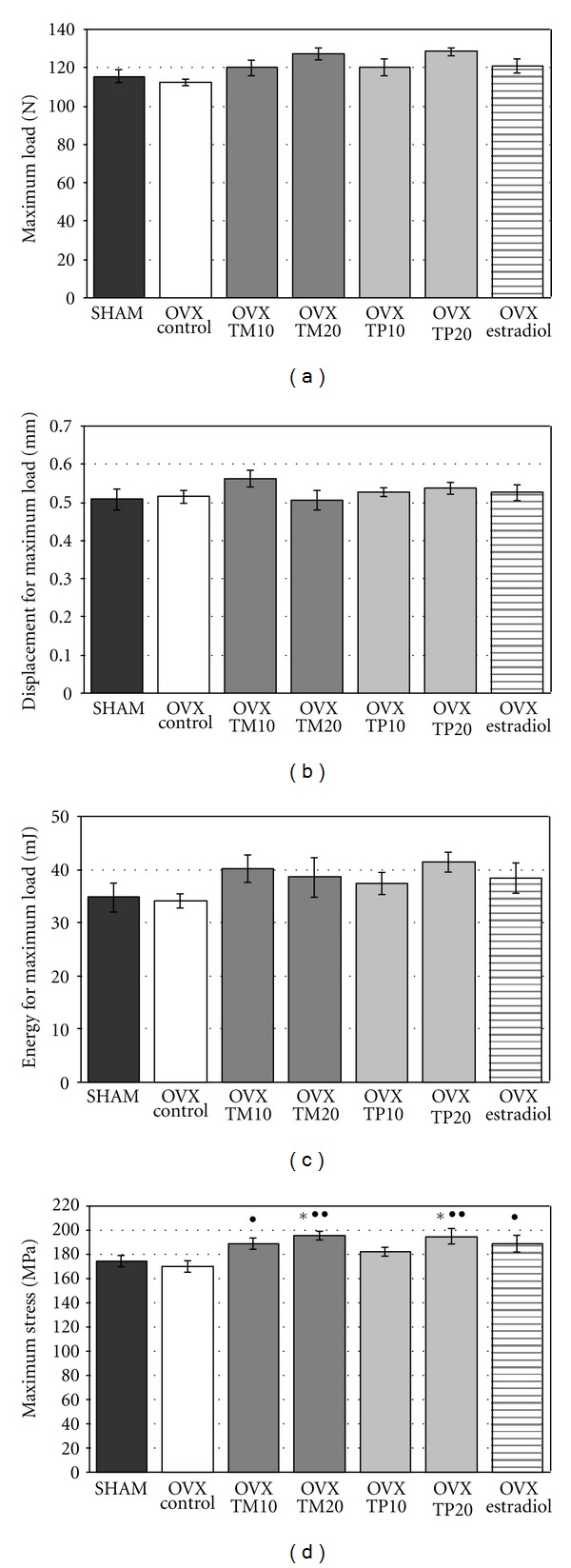
Effects of *Trifolium medium* L. (TM) and *Trifolium pratense* L. (TP) extracts on mechanical properties of the femoral diaphysis (the maximum load point) in ovariectomized (OVX) rats. The lyophilized extracts from TM or TP at doses corresponding to 10 and 20 mg/kg of isoflavone aglycones daily (TM10, TP10 and TM20, TP20, resp.), or estradiol (0.2 mg/kg daily), were administered orally to OVX rats for 4 weeks. Results are presented as the mean ± SEM (*n* = 9-10). One-way ANOVA followed by Duncan's test was used for evaluation of the significance of the results. *P < 0.05—significantly different from sham-operated control rats (SHAM). ^•^ *P* < 0.05,^ ••^ *P* < 0.01—significantly different from OVX control rats.

Mechanical properties of the femoral diaphysis were not affected by estrogen deficiency. Young's modulus, as well as extrinsic and intrinsic parameters determined for the maximum load (Figure 1) and the fracture point load (Table 4) in ovariectomized control rats, did not differ from those of the sham-operated control rats.

Administration of the extracts from both *Trifolium* species increased the strength of the femoral diaphysis in the ovariectomized rats. The maximum load and the energy for the maximum load tended to increase after administration of both isoflavone doses, and the maximum stress was significantly increased after administration of *T. medium* at both doses and *T. pratense* at the higher dose, when compared with the ovariectomized control rats (Figure 1). Similarly, the load and stress for the fracture point significantly increased, in relation to the ovariectomized control rats, after administration of the higher isoflavone dose. There was no significant effect of the extracts on the Young's modulus of the femoral diaphysis (Table 4).

After administration of estradiol, the stress for the maximum load and for the fracture load significantly increased, in comparison with the ovariectomized control rats; however the effect was slighter than that observed after administration of the higher *Trifolium* extract doses. 

### 3.5. Mechanical Properties of the Femoral Neck

**Figure 2:**
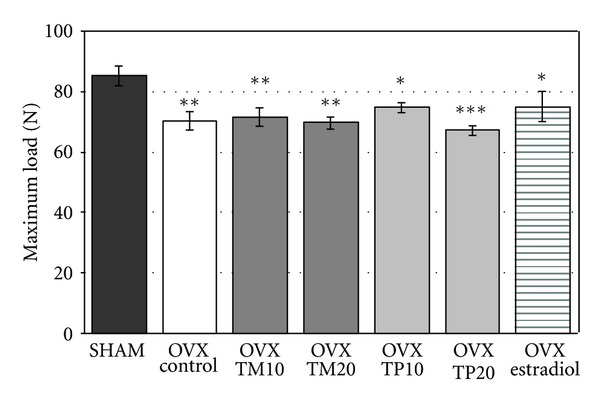
Effects of *Trifolium medium* L. (TM) and *Trifolium pratense* L. (TP) extracts on the strength of the femoral neck in ovariectomized (OVX) rats. The lyophilized extracts from TM or TP at doses corresponding to 10 and 20 mg/kg of isoflavone aglycones daily (TM10, TP10 and TM20, TP20, resp.), or estradiol (0.2 mg/kg daily), were administered orally to OVX rats for 4 weeks. Results are presented as the mean ± SEM (*n* = 9-10). One-way ANOVA followed by Duncan's test was used for evaluation of the significance of the results, *P < 0,05.**P < 0.01, ***P < 0.001—significantly different from sham-operated control rats (SHAM).

Estrogen deficiency caused a statistically significant decrease in the strength of the femoral neck in the ovariectomized control rats in relation to the sham-operated control rats (Figure 2).

Administration of the *Trifolium* extracts at both isoflavone doses did not affect the maximum load sustained by the femoral neck of the ovariectomized rats. Also supplementation of estradiol to the ovariectomized rats did not significantly affect the strength of the femoral neck.

### 3.6. Histomorphometric Parameters of the Femur

 Although the transverse growth in the femur of the ovariectomized control rats was slightly increased in comparison with the sham-operated rats, the transverse cross-section area of the cortical bone and of the whole diaphysis were not significantly affected (Table 5). However, there was a significant increase in the ratio of the transverse cross-section area of the marrow cavity to the area of the whole diaphysis, indicating the increased cortical bone resorption. The width of trabeculae in the femoral epiphysis and metaphysis significantly decreased in the ovariectomized control rats, indicating a possibility of increased cancellous bone resorption.

 The *Trifolium *extracts did not affect the histomorphometric parameters of the femoral diaphysis of the ovariectomized rats but increased the width of epiphyseal trabeculae. Only *T. pratense* extract significantly increased the width of metaphyseal trabeculae.

 Administration of estradiol significantly counteracted the effect of estrogen deficiency on the transverse cross-section marrow cavity/diaphysis area ratio in the diaphysis and the width of trabeculae in epiphysis and metaphysis of the femur.

### 3.7. Serum Biochemical Bone Turnover Markers

Estrogen deficiency increased the serum level of the biochemical marker of bone resorption (RatLaps) and tended to increase the marker of bone formation (osteocalcin) in comparison with the sham-operated controls (Table 6).

The *Trifolium *extracts did not affect the levels of the serum markers of bone turnover in ovariectomized rats.

Supplementation of the ovariectomized rats with estradiol normalized the levels of bone turnover markers.

## 4. Discussion

Administration of the *Trifolium* extracts significantly affected the skeletal system of ovariectomized rats, counteracting some changes induced by estrogen deficiency, and also inducing changes which seemed to be unrelated to typical estrogenic effects in rats with decreased estrogen levels. There were some differences between the effects of *Trifolium* extracts and those of estradiol, and also differences between the effects of *T. medium *and* T. pratense*.

The data on the skeletal effects of *T. pratense* extracts are very limited, but there are numerous reports on skeletal effects of soy isoflavones. The most abundant soy isoflavones (aglycones) are genistein and daidzein, and the main *Trifolium* isoflavones, biochanin A and formononetin, are metabolized to genistein and daidzein, respectively, in mammalian organisms [27]. Nevertheless, the results of studies on soy product effects on the skeletal system in humans are inconsistent [28–32]. Interestingly, a recent study on the effects of dietary phytoestrogens on bone density performed in a European population indicated an independent association between bone density and formononetin in postmenopausal women and biochanin A in men [33]. However, the most important data on the isoflavone effects on fracture risk are still missing [1, 34]. 

So far, differential effects of particular red clover isoflavones on the skeletal system were demonstrated in experimental *in vivo* studies on biochanin A [35], formononetin [36, 37], genistein [35, 38–46], daidzein [35, 46], and an active metabolite of daidzein—equol [43–46]. However, in aforementioned studies, free isoflavone aglycones were used, whereas in the plants, and in plant extracts (also those examined in our study), isoflavones occur mainly as glycosides. 

The present study was carried out using a model of 3-month-old ovariectomized rats, developing osteoporotic changes due to estrogen deficiency. *Trifolium* extracts were administered to the rats for 4 weeks, starting a week after the ovariectomy. A 4-week period of administration was long enough to observe skeletal effects of substances of plant origin in our previous studies [25, 41, 47, 48].

Estrogen deficiency induced characteristic osteoporotic changes in the ovariectomized control rats. Supplementation with estradiol, in most parameters, counteracted the effects of estrogen deficiency: the worsening of the tibial strength, the changes in bone histomorphometric parameters, and increases in bone turnover markers. Moreover, some parameters of the femoral diaphysis improved.

The *Trifolium *extracts did not counteract the effects of estrogen deficiency so consistently as did estradiol. Only *T. pratense* extract increased the strength of the tibial metaphysis (this effect was weaker than that of estradiol). *T. pratense* extract also exerted stronger effect on a cancellous bone histomorphometric parameter (metaphyseal trabeculae width). The reason for the stronger estrogenic activity of the *T. pratense* extract may be speculated. It has been proposed that the estrogenic activity of isoflavones is connected with their metabolism to equol [46, 49, 50]. For the formation of equol, exclusively intestinal microflora is responsible [49]. From among the *Trifolium* isoflavones, formononetin and daidzein can be metabolized to equol, and biochanin A and genistein cannot [49, 50]. Total content of formononetin and daidzein in *T. medium* and *T. pratense* extracts was very similar. However, the *T. pratense* extract, containing smaller amount of isoflavone aglycones, had to be administered at higher doses in order to secure the same isoflavone aglycone intake. Bigger amount of the administered *T. pratense *extract, characteristic of high carbohydrate content, might have contributed to the creation of better environment that stimulated the growth of bacteria responsible for the metabolism of isoflavone glycosides, followed by higher equol production. It has been observed that bioavailability of soy isoflavones depends, among others, on the activity of intestinal bacteria, and a diet rich in carbohydrates may stimulate equol production [50].

Apart from the effects on the cancellous bone, similar to those observed for estradiol, both *Trifolium *extracts exerted other skeletal effects in estrogen-deficient rats: a significant increase in the strength of the compact bone of the femoral diaphysis and increases in the calcium and phosphorus content in the bone mineral of the vertebra and tibia, but not of the femur. The improvement in the strength of the cortical bone of the femoral diaphysis, induced by the *Trifolium *extracts, is consistent with a recent peripheral quantitative computed tomography study of Shedd-Wise et al. [51], who reported that, in postmenopausal women treated with soy isoflavones, some protective effect on cortical bone was observed.

It may be speculated that some skeletal effects of the *Trifolium* extracts may be attributed, at least in part, to other plant constituents, like other flavonoids and phenolic acids; some of phenolic acids have been reported to favorably affect the skeletal system of ovariectomized rats [47, 52]. In our recent study we have observed that caffeic and chlorogenic acids at high doses slightly increased the strength of cortical bone of the femoral diaphysis only [53]. The mechanism of action of the extracts may be connected with antioxidant activity of polyphenolic compounds, including isoflavones. The role of oxidative stress in the development of osteoporosis gains growing attention [54]. 

The increasing effects of the *Trifolium* extracts on the calcium and phosphorus content in the bone mineral of the tibia and vertebra are surprising. The changes were not accompanied with the increase of the mineral substances (ash) content in the bones. The exact character of changes in the mineral chemical structure, as well as the meaning of the changes for the bone quality, is not known. Significant increases in the calcium and phosphorus content were not observed in the femurs in which thick diaphysis (compact bone) strongly contributes to the bone mass. The *Trifolium *extracts improved mechanical quality of the femoral diaphysis. On the other hand, there was much less effect or no effect of the extracts on the mechanical properties of cancellous bone of the tibia. It is possible that antiresorptive effect in cancellous bone (increases in the trabeculae width observed in the femur) is balanced by in fact unfavorable effect of the structural changes in bone mineral, which in consequence does not allow improving bone strength. The issue needs further investigations. 

It may only be speculated that the increase in the calcium content may be the result of a high intake of sugars present in the *Trifolium* extracts. Some of sugars might have reached the colon and have been fermented by intestinal bacteria, reducing pH of the environment and increasing calcium absorption. So far, such mechanism was proposed for inulin-type fructans, which were reported to increase calcium absorption [55]. Increased calcium absorption was accompanied with increased mineral concentrations in bones of rats fed a diet containing fructooligosaccharides [56]. *Trifolium pratense* does not contain fructans but is rich in other nonstructural carbohydrates [57]. 

Contrary to estradiol, the extracts did not affect the uterus mass. Moreover, there were no effects on the body mass gain and total cholesterol level, indicating the possibility that the *Trifolium* phytoestrogens did not affect all estrogenic targets in the body. The lack of significant effect on uterine mass is consistent with an experimental study on rabbits [15]. In fact, relative binding affinity of particular *Trifolium* isoflavones to the rat uterine estrogen receptors was much lower than that of estradiol [58]. The lack of significant effects on the uterus has been attributed to their preferential binding to estrogen receptor *β*, which is less expressed in the uterus [15]. Taking into consideration estrogenic effects on bones, results of the present study support the notion that *Trifolium* phytoestrogens may act as SERMs. It should be stated, however, that higher doses of red clover extract resulted in estrogenic effects, increasing the uterus mass and differentiated vaginal cells in ovariectomized rats [59]. 

Some skeletal effects of the extracts observed in the present study in rats may not be observed in humans due to differential pattern of isoflavone metabolism by intestinal bacterial flora. Probably due to differences in the intestinal bacterial flora, humans divide into equol producers, and equol nonproducers [50, 60]. Rats, with their large cecum and abundant microflora [49], are much more capable to produce equol than humans [61]. Moreover, different compounds of phytoestrogenic plant extracts undergo metabolism dependent on gut microflora and human enzymes, and there is great interindividual variation concerning the metabolism [27, 50]. Little is known on the extent to which different metabolites, exerting agonistic or antagonistic activity towards *α* or *β* estrogen receptors, are formed after consumption of isoflavone-rich foods [27]. Intake of phytoestrogens may be connected not only with health benefits. Except affecting estrogen receptors, they may interact with other biological systems, as comprehensively reviewed by Leclercq et al. [62], and exert serious adverse effects [63]. Estrogenic effects of the *Trifolium* extracts on different targets, including those connected with blood coagulation and risk of stroke or venous thromboembolism, are possible and need further studies. 

The present study has some limitations. One of them is a single study period (4 weeks), which may be considered as a relatively short one for testing the effects of dietary supplements on estrogen-deficiency model in rats. It is possible that longer time of the *Trifolium *extract administration would allow to observe more of their estrogenic effects, including those on bone turnover markers. Another limitation of the study is that it concerns only the effects of the extracts; parallel examination of their main components would enable pointing the compounds responsible for their skeletal activity. This issue requires further studies.

In conclusion, the effects of *T. medium* and *T. pratense* extracts on the skeletal system of estrogen-deficient rats differed from those exerted by estradiol, indicating more complex mechanism of action. Skeletal effects of *T. medium* extract seemed to be less similar to those exerted by estradiol than the effects of *T. pratense* extract. It is possible that other than isoflavone constituents of extracts contributed to their effects on the skeletal system. 

## Figures and Tables

**Table 1:** Effects of *Trifolium medium* L. (TM) and *Trifolium pratense* L. (TP) extracts on the body mass gain, mass of estrogen-dependent organs, and serum levels of estradiol and total cholesterol in ovariectomized (OVX) rats.

Parameters	Sham- operated rats	OVX rats					
		Control	TM10	TM20	TP10	TP20	Estradiol
Final body mass (g)	234.8 ± 3.6	263.1 ± 4.5***	268.3 ± 4.2***	274.8 ± 4.8***	263.9 ± 3.9***	265.2 ± 4.6***	250.6 ± 5.0*
Body mass gain after 28 days (g)	16.5 ± 2.4	39.0 ± 2.5***	42.1 ± 2.3***	43.8 ± 3.6***	38.3 ± 2.0***	40.7 ± 3.3***	25.6 ± 2.9^∗ ●●^
Uterus mass (mg/100 g of body mass)	235.66 ± 27.70	35.09 ± 1.81***	35.64 ± 1.60***	36.03 ± 1.41***	39.39 ± 1.67***	37.19 ± 1.79***	67.31 ± 4.89^∗∗∗ ●●●^
Thymus mass (mg/100 g of body mass)	130.07 ± 11.12	196.83 ± 9.25**	203.63 ± 21.41**	199.37 ± 9.66***	201.27 ± 13.81**	196.57 ± 11.35*	181.74 ± 15.74**
Total cholesterol (mg/100 mL)	49.93 ± 2.90	69.11 ± 2.83***	68.88 ± 3.61***	65.66 ± 2.62**	68.23 ± 3.17***	66.13 ± 3.30**	56.19 ± 3.81^●^
Estradiol (pg/mL)	20.64 ± 3.05	10.37 ± 0.80*	9.83 ± 0.63**	11.68 ± 0.61	9.34 ± 0.44**	10.95 ± 1.07*	13.84 ± 1.26

The lyophilized extracts from TM or TP at doses corresponding to 10 and 20 mg/kg of isoflavone aglycones daily (TM10, TP10 and TM20, TP20, resp.), or estradiol (0.2 mg/kg daily), were administered orally to OVX rats for 4 weeks. Results are presented as the mean ± SEM (*n* = 9-10). One-way ANOVA followed by Duncan's test or, when appropriate, Kruskal-Wallis ANOVA followed by Mann-Whitney *U* test was used for evaluation of the significance of the results. *P < 0.05, **P < 0.01, ***P < 0.001—significantly different from sham-operated control rats. ^●^ *P* < 0.05, ^●●^ *P* < 0.01, ^●●●^ *P* < 0.001—significantly different from OVX control rats.

**Table 2:** Effects of *Trifolium medium* L. (TM) and *Trifolium pratense* L. (TP) extracts on bone mass and mineralization in ovariectomized (OVX) rats.

Parameters		Sham- operated rats	OVX rats					
			Control	TM10	TM20	TP10	TP20	Estradiol
Bone mass/body mass ratio (mg/100 g of body mass)	Femur	296.04 ± 3.89	259.58 ± 4.14***	262.10 ± 4.81***	254.99 ± 5.24***	258.66 ± 4.24***	267.53 ± 6.82***	271.58 ± 4.11***
	Tibia	225.87 ± 3.06	198.61 ± 2.43***	193.37 ± 3.14***	192.83 ± 2.74***	200.52 ± 2.80***	200.79 ± 3.72***	202.79 ± 3.40***
	L-4 vertebra	80.48 ± 1.46	72.80 ± 2.18**	68.43 ± 1.04***	68.52 ± 1.11***	73.18 ± 0.94**	72.46 ± 1.65**	75.65 ± 2.03

Mineral mass/bone mass ratio (mg/100 mg of bone mass)	Femur	45.74 ± 0.34	43.92 ± 0.41**	42.59 ± 0.47***	43.17 ± 0.38***	44.42 ± 0.43	43.47 ± 0.56**	44.48 ± 0.47*
	Tibia	46.38 ± 0.56	44.37 ± 0.41**	43.99 ± 0.27***	44.38 ± 0.39**	44.42 ± 0.42**	45.21 ± 0.36	45.45 ± 0.55
	L-4 vertebra	44.32 ± 0.48	42.29 ± 0.77	42.80 ± 0.51	42.64 ± 0.35	43.11 ± 0.45	42.30 ± 0.56	42.92 ± 0.62

Calcium content (mg/g of mineral mass)	Femur	382.79 ± 4.77	379.61 ± 4.73	395.65 ± 5.20	396.39 ± 5.25	394.26 ± 4.06	395.70 ± 6.08	391.82 ± 5.48
	Tibia	397.83 ± 3.93	406.06 ± 9.05	443.56 ± 10.99^∗∗●^	445.64 ± 9.88^∗∗∗●●^	439.05 ± 9.18^∗∗ ●^	446.67 ± 11.49^∗∗●^	425.57 ± 14.43
	L-4 vertebra	409.72 ± 7.67	409.35 ± 5.82	446.48 ± 6.59^∗ ●^	444.44 ± 12.09^∗●^	451.76 ± 9.76^∗∗●●^	453.69 ± 12.65^∗∗●●^	427.06 ± 12.12

Phosphorus content (mg/g of mineral mass)	Femur	161.50 ± 1.73	160.89 ± 1.60	163.82 ± 1.29	163.92 ± 1.51	164.47 ± 1.38	164.41 ± 1.32	162.48 ± 3.25
	Tibia	154.36 ± 2.17	153.32 ± 2.65	168.77 ± 5.30^∗ ●^	169.92 ± 5.29^∗ ●^	168.61 ± 3.99^∗ ●^	168.28 ± 4.36^∗ ●^	153.65 ± 3.31
	L-4 vertebra	165.01 ± 2.07	163.93 ± 2.91	178.55 ± 4.93^∗ ●^	177.09 ± 3.77^∗ ●^	176.66 ± 2.97^∗ ●^	178.18 ± 5.12^∗ ●^	171.48 ± 3.45

The lyophilized extracts from TM or TP at doses corresponding to 10 and 20 mg/kg of isoflavone aglycones daily (TM10, TP10 and TM20, TP20, resp.), or estradiol (0.2 mg/kg daily), were administered orally to OVX rats for 4 weeks. Results are presented as the mean ± SEM (*n* = 9-10). One-way ANOVA followed by Duncan's test or, when appropriate, Kruskal-Wallis ANOVA followed by Mann-Whitney *U* test was used for evaluation of the significance of the results. *P < 0.05, **P < 0.01, ***P < 0.001—significantly different from sham-operated control rats. ^●^ *P* < 0.05, ^●●^ *P* < 0.01—significantly different from OVX control rats.

**Table 3:** Effects of *Trifolium medium* L. (TM) and *Trifolium pratense* L. (TP) extracts on mechanical properties of the tibial metaphysis in ovariectomized (OVX) rats.

Parameters	Sham- operated rats	OVX rats					
		Control	TM10	TM20	TP10	TP20	Estradiol
Maximum load (*N*)	120.63 ± 3.95	66.85 ± 3.56***	70.02 ± 2.53***	69.76 ± 2.41***	79.66 ± 2.86^∗∗∗ ●●^	77.12 ± 2.86^∗∗∗ ●^	90.38 ± 3.08^∗∗∗ ●●●^
Displacement for maximum load (mm)	0.84 ± 0.06	0.90 ± 0.07	0.83 ± 0.04	0.88 ± 0.02	0.82 ± 0.03	0.89 ± 0.06	0.82 ± 0.05
Energy for maximum load (mJ)	52.02 ± 4.36	39.20 ± 4.61**	33.52 ± 2.33***	36.49 ± 1.61**	36.58 ± 2.67**	37.77 ± 2.86**	39.93 ± 2.91**
Maximum stress (MPa)	103.82 ± 4.23	54.82 ± 4.38***	56.34 ± 3.61***	55.81 ± 2.22***	60.05 ± 2.69***	59.65 ± 2.77***	73.74 ± 4.07^∗∗∗ ●●●^
Young's modulus (MPa)	3410.78 ± 237.74	2318.16 ± 182.21**	2246.02 ± 147.56**	2248.36 ± 117.87***	2149.64 ± 178.89***	2417.34 ± 140.06***	2770.31 ± 286.73

The lyophilized extracts from TM or TP at doses corresponding to 10 and 20 mg/kg of isoflavone aglycones daily (TM10, TP10 and TM20, TP20, resp.), or estradiol (0.2 mg/kg daily), were administered orally to OVX rats for 4 weeks. Results are presented as the mean ± SEM (*n* = 9-10). One-way ANOVA followed by Duncan's test or, when appropriate, Kruskal-Wallis ANOVA followed by Mann-Whitney *U* test was used for evaluation of the significance of the results. **P < 0.01, ***P < 0.001—significantly different from sham-operated control rats. ^●^ *P* < 0.05, ^●●^ *P* < 0.01, ^●●●^ *P* < 0.001—significantly different from OVX control rats.

**Table 4:** Effects of *Trifolium medium* L. (TM) and *Trifolium pratense* L. (TP) extracts on mechanical properties of the femoral diaphysis (parameters for the fracture point and Young's modulus) in ovariectomized (OVX) rats.

Parameters	Sham- operated rats	OVX rats					
		Control	TM10	TM20	TP10	TP20	Estradiol
Fracture load (*N*)	114.56 ± 3.94	111.98 ± 2.33	116.01 ± 4.44	125.82 ± 3.44^●^	116.84 ± 4.00	127.66 ± 2.66^∗ ●●^	120.15 ± 3.80
Displacement for fracture load (mm)	0.52 ± 0.03	0.52 ± 0.02	0.61 ± 0.04	0.52 ± 0.03	0.55 ± 0.02	0.56 ± 0.02	0.54 ± 0.02
Energy for fracture load (mJ)	35.69 ± 2.75	34.71 ± 1.55	46.36 ± 5.00	40.07 ± 4.29	40.04 ± 2.89	43.73 ± 2.66	40.56 ± 3.58
Stress for fracture load (MPa)	172.68 ± 5.76	169.16 ± 5.34	182.65 ± 6.06	193.14 ± 4.41^∗ ●●^	178.01 ± 3.63	193.34 ± 6.89^∗ ●●^	187.79 ± 7.14^●^
Young's modulus (MPa)	9368.24 ± 430.74	8679.70 ± 689.37	9612.18 ± 499.99	9651.65 ± 412.61	9356.92 ± 148.39	8759.22 ± 1028.53	9687.52 ± 654.88

The lyophilized extracts from TM or TP at doses corresponding to 10 and 20 mg/kg of isoflavone aglycones daily (TM10, TP10 and TM20, TP20, resp.), or estradiol (0.2 mg/kg daily), were administered orally to OVX rats for 4 weeks. Results are presented as the mean ± SEM (*n* = 9-10). One-way ANOVA followed by Duncan's test or, when appropriate, Kruskal-Wallis ANOVA followed by Mann-Whitney *U* test was used for evaluation of the significance of the results. *P < 0.05—significantly different from sham-operated control rats. ^●^ *P* < 0.05. ^●●^ *P* < 0.01—significantly different from OVX control rats.

**Table 5:** Effects of *Trifolium medium* L. (TM) and *Trifolium pratense* L. (TP) extracts on histomorphometric parameters of the femur in ovariectomized (OVX) rats.

Parameters		Sham- operated rats	OVX rats					
			Control	TM10	TM20	TP10	TP20	Estradiol
Transverse cross-section area (mm^2^)	Cortical bone	5.25 ± 0.08	5.13 ± 0.08	5.12 ± 0.10	5.31 ± 0.09	5.22 ± 0.06	5.28 ± 0.10	5.26 ± 0.11
	Marrow cavity	2.65 ± 0.08	2.82 ± 0.05	2.82 ± 0.08	2.75 ± 0.14	2.86 ± 0.06	2.77 ± 0.07	2.55 ± 0.10
	Whole diaphysis	7.90 ± 0.10	7.96 ± 0.12	7.94 ± 0.16	8.06 ± 0.15	8.08 ± 0.10	8.05 ± 0.10	7.82 ± 0.19

Transverse cross-section marrow cavity/diaphysis area ratio		0.335 ± 0.008	0.355 ± 0.003*	0.355 ± 0.006	0.339 ± 0.012	0.354 ± 0.005*	0.344 ± 0.009	0.326 ± 0.007^●●^

Transverse growth (*μ*m)	Periosteal	38.68 ± 2.50	43.70 ± 2.99	40.14 ± 2.84	40.01 ± 1.98	39.65 ± 0.88	37.75 ± 2.24	38.37 ± 3.91
	Endosteal	28.54 ± 2.99	33.62 ± 2.22	31.47 ± 2.45	29.70 ± 2.90	31.89 ± 1.81	29.75 ± 1.08	29.74 ± 1.67

Width of trabeculae (*μ*m)	Epiphysis	58.76 ± 0.79	53.47 ± 1.09**	57.03 ± 0.73^●^	57.28 ± 0.67^●^	57.85 ± 1.26^●^	59.42 ± 1.35^●●^	60.53 ± 1.49^●●^
	Metaphysis	36.04 ± 0.59	32.98 ± 0.86**	34.54 ± 1.03	35.00 ± 0.80	36.23 ± 0.70^●^	37.46 ± 1.35^●●^	38.49 ± 1.78^●^

The lyophilized extracts from TM or TP at doses corresponding to 10 and 20 mg/kg of isoflavone aglycones daily (TM10, TP10 and TM20, TP20, resp.), or estradiol (0.2 mg/kg daily), were administered orally to OVX rats for 4 weeks. Results are presented as the mean ± SEM (*n* = 9-10). One-way ANOVA followed by Duncan's test or, when appropriate, Kruskal-Wallis ANOVA followed by Mann-Whitney *U* test was used for evaluation of the significance of the results. *P < 0.05, **P < 0.01—significantly different from sham-operated control rats. ^●^ *P* < 0.05, ^●●^ *P* < 0.01—significantly different from OVX control rats.

**Table 6:** Effects of *Trifolium medium* L. (TM) and *Trifolium pratense* L. (TP) extracts on the serum bone turnover markers in ovariectomized (OVX) rats.

Parameters	Sham- operated rats	OVX rats					
		Control	TM10	TM20	TP10	TP20	Estradiol
Osteocalcin (ng/mL)	238.30 ± 27.70	325.47 ± 26.72	327.36 ± 28.13	344.84 ± 25.61	344.84 ± 38.73	332.50 ± 39.91	254.33 ± 17.13
RatLaps (ng/mL)	20.88 ± 1.82	30.89 ± 1.44**	30.79 ± 1.99**	30.74 ± 1.93**	32.99 ± 1.78***	28.59 ± 2.09*	23.39 ± 2.80^●^

The lyophilized extracts from TM or TP at doses corresponding to 10 and 20 mg/kg of isoflavone aglycones daily (TM10, TP10 and TM20, TP20, resp.), or estradiol (0.2 mg/kg daily), were administered orally to OVX rats for 4 weeks. Results are presented as the mean ± SEM (*n* = 9-10). One-way ANOVA followed by Duncan's test was used for evaluation of the significance of the results. *P < 0.05, **P < 0.01, ***P < 0.001—significantly different from sham-operated control rats. ^●^ *P* < 0.05—significantly different from OVX control rats.

## References

[1] Lagari VS, Levis S (2010). Phytoestrogens and bone health. *Current Opinion in Endocrinology, Diabetes and Obesity*.

[2] Oseni T, Patel R, Pyle J, Jordan VC (2008). Selective estrogen receptor modulators and phytoestrogens. *Planta Medica*.

[3] Zgórka G (2011). Studies on phytoestrogenic and nonphytoestrogenic compounds in *Trifolium incarnatum* L. and other clover species using pressurized liquid extraction and high performance column liquid chromatography with photodiode-array and fluorescence detection. *Journal of AOAC International*.

[4] Sabudak T, Guler N (2009). *Trifolium* L.—a review on its phytochemical and pharmacological profile. *Phytotherapy Research*.

[5] Coon JT, Pittler MH, Ernst E (2007). *Trifolium pratense* isoflavones in the treatment of menopausal hot flushes: a systematic review and meta-analysis. *Phytomedicine*.

[6] Geller SE, Shulman LP, van Breemen RB (2009). Safety and efficacy of black cohosh and red clover for the management of vasomotor symptoms: a randomized controlled trial. *Menopause*.

[7] del Giorno C, da Fonseca AM, Bagnoli VR, de Assis JS, Soares JM, Baracat EC (2010). Effects of *Trifolium pratense* on the climacteric and sexual symptoms in postmenopausal women. *Revista da Associação Médica Brasileira*.

[8] Lipovac M, Chedraui P, Gruenhut C (2012). The effect of red clover isoflavone supplementation over vasomotor and menopausal symptoms in postmenopausal women. *Gynecological Endocrinology*.

[9] Panay N (2011). Taking an integrated approach: managing women with phytoestrogens. *Climacteric*.

[10] Knudson Schult TM, Ensrud KE, Blackwell T, Ettinger B, Wallace R, Tice JA (2004). Effect of isoflavones on lipids and bone turnover markers in menopausal women. *Maturitas*.

[11] Weaver CM, Martin BR, Jackson GS (2009). Antiresorptive effects of phytoestrogen supplements compared with estradiol or risedronate in postmenopausal women using ^41^Ca methodology. *The Journal of Clinical Endocrinology and Metabolism*.

[12] Clifton-Bligh PB, Baber RJ, Fulcher GR, Nery ML, Moreton T (2001). The effect of isoflavones extracted from red clover (Rimostil) on lipid and bone metabolism. *Menopause*.

[13] Atkinson C, Compston JE, Day NE, Dowsett M, Bingham SA (2004). The effects of phytoestrogen isoflavones on bone density in women: a double-blind, randomized, placebo-controlled trial. *The American Journal of Clinical Nutrition*.

[14] Occhiuto F, De Pasquale R, Guglielmo G (2007). Effects of phytoestrogenic isoflavones from red clover (*Trifolium pratense* L.) on experimental osteoporosis. *Phytotherapy Research*.

[15] Adaikan PG, Srilatha B, Wheat AJ (2009). Efficacy of red clover isoflavones in the menopausal rabbit model. *Fertility and Sterility*.

[16] Kawakita S, Marotta F, Naito Y (2009). Effect of an isoflavones-containing red clover preparation and alkaline supplementation on bone metabolism in ovariectomized rats. *Clinical Interventions in Aging*.

[17] Zgórka G (2009). Pressurized liquid extraction versus other extraction techniques in micropreparative isolation of pharmacologically active isoflavones from *Trifolium* L. species. *Talanta*.

[18] Bisby FA, Zarucchi JL, Schrire BD, Roskov YR, White RJ (2000). *ILDIS World Database of Legumes*.

[19] Zoric L, Merkulov L, Lukovic J, Boza P (2012). Comparative analysis of qualitative anatomical characters of *Trifolium* L. (Fabaceae) and their taxonomic implications: preliminary results. *Plant Systematics and Evolution*.

[20] Turner CH, Burr DB (1993). Basic biomechanical measurements of bone: a tutorial. *Bone*.

[21] Stürmer EK, Seidlová-Wuttke D, Sehmisch S (2006). Standardized bending and breaking test for the normal and osteoporotic metaphyseal tibias of the rat: effect of estradiol, testosterone, and raloxifene. *Journal of Bone and Mineral Research*.

[22] Folwarczna J, Nowińska B, Śliwiński L, Pytlik M, Cegieła U, Betka A (2011). Fenoterol did not enhance glucocorticoid-induced skeletal changes in male rats. *Acta Biochimica Polonica*.

[23] Kiebzak GM, Smith R, Gundberg CC, Howe JC, Sacktor B (1988). Bone status of senescent male rats: chemical, morphometric, and mechanical analysis. *Journal of Bone and Mineral Research*.

[24] Pytlik M, Folwarczna J, Janiec W (2004). Effects of doxycycline on mechanical properties of bones in rats with ovariectomy-induced osteopenia. *Calcified Tissue International*.

[25] Cegieła U, Pytlik M, Janiec W (2000). Effects of *α*-escin on histomorphometrical parameters of long bones in rats with experimental post-steroid osteopenia. *Polish Journal of Pharmacology*.

[26] Folwarczna J, Śliwiński L, Cegieła U (2007). Raloxifene similarly affects the skeletal system of male and ovariectomized female rats. *Pharmacological Reports*.

[27] Pfitscher A, Reiter E, Jungbauer A (2008). Receptor binding and transactivation activities of red clover isoflavones and their metabolites. *Journal of Steroid Biochemistry and Molecular Biology*.

[28] Ma DF, Qin LQ, Wang PY, Katoh R (2008). Soy isoflavone intake increases bone mineral density in the spine of menopausal women: meta-analysis of randomized controlled trials. *Clinical Nutrition*.

[29] Ma DF, Qin LQ, Wang PY, Katoh R (2008). Soy isoflavone intake inhibits bone resorption and stimulates bone formation in menopausal women: meta-analysis of randomized controlled trials. *European Journal of Clinical Nutrition*.

[30] Liu J, Ho SC, Su YX, Chen WQ, Zhang CX, Chen YM (2009). Effect of long-term intervention of soy isoflavones on bone mineral density in women: a meta-analysis of randomized controlled trials. *Bone*.

[31] Ricci E, Cipriani S, Chiaffarino F, Malvezzi M, Parazzini F (2010). Soy isoflavones and bone mineral density in perimenopausal and postmenopausal western women: a systematic review and meta-analysis of randomized controlled trials. *Journal of Women’s Health*.

[32] Taku K, Melby MK, Nishi N, Omori T, Kurzer MS (2011). Soy isoflavones for osteoporosis: an evidence-based approach. *Maturitas*.

[33] Kuhnle GGC, Ward HA, Vogiatzoglou A (2011). Association between dietary phyto-oestrogens and bone density in men and postmenopausal women. *The British Journal of Nutrition*.

[34] Castelo-Branco C, Cancelo Hidalgo MJ (2011). Isoflavones: effects on bone health. *Climacteric*.

[35] Somjen D, Katzburg S, Kohen F, Gayer B, Livne E (2008). Daidzein but not other phytoestrogens preserves bone architecture in ovariectomized female rats in vivo. *Journal of Cellular Biochemistry*.

[36] Gautam AK, Bhargavan B, Tyagi AM (2011). Differential effects of formononetin and cladrin on osteoblast function, peak bone mass achievement and bioavailability in rats. *The Journal of Nutritional Biochemistry*.

[37] Ha H, Lee HY, Lee JH (2010). Formononetin prevents ovariectomy-induced bone loss in rats. *Archives of Pharmacal Research*.

[38] Anderson JJB, Ambrose WW, Garner SC (1998). Biphasic effects of genistein on bone tissue in the ovariectomized, lactating rat model. *Proceedings of the Society for Experimental Biology and Medicine*.

[39] Fanti P, Monier-Faugere MC, Geng Z (1998). The phytoestrogen genistein reduces bone loss in short-term ovariectomized rats. *Osteoporosis International*.

[40] Bitto A, Burnett BP, Polito F (2008). Effects of genistein aglycone in osteoporotic, ovariectomized rats: a comparison with alendronate, raloxifene and oestradiol. *British Journal of Pharmacology*.

[41] Śliwiński L, Folwarczna J, Nowińska B (2009). A comparative study of the effects of genistein, estradiol and raloxifene on the murine skeletal system. *Acta Biochimica Polonica*.

[42] Bitto A, Marini H, Burnett BP (2011). Genistein aglycone effect on bone loss is not enhanced by supplemental calcium and vitamin D3: a dose ranging experimental study. *Phytomedicine*.

[43] Tezval M, Sehmisch S, Seidlová-Wuttke D (2010). Changes in the histomorphometric and biomechanical properties of the proximal femur of ovariectomized rat after treatment with the phytoestrogens genistein and equol. *Planta Medica*.

[44] Sehmisch S, Erren M, Kolios L (2010). Effects of isoflavones equol and genistein on bone quality in a rat osteopenia model. *Phytotherapy Research*.

[45] Sehmisch S, Uffenorde J, Maehlmeyer S (2010). Evaluation of bone quality and quantity in osteoporotic mice—the effects of genistein and equol. *Phytomedicine*.

[46] Mathey J, Mardon J, Fokialakis N (2007). Modulation of soy isoflavones bioavailability and subsequent effects on bone health in ovariectomized rats: the case for equol. *Osteoporosis International*.

[47] Folwarczna J, Zych M, Burczyk J, Trzeciak H, Trzeciak HI (2009). Effects of natural phenolic acids on the skeletal system of ovariectomized rats. *Planta Medica*.

[48] Folwarczna J, Zych M, Trzeciak HI (2010). Effects of curcumin on the skeletal system in rats. *Pharmacological Reports*.

[49] Setchell KDR, Brown NM, Lydeking-Olsen E (2002). The clinical importance of the metabolite equol—a clue to the effectiveness of soy and its isoflavones. *The Journal of Nutrition*.

[50] Yuan JP, Wang JH, Liu X (2007). Metabolism of dietary soy isoflavones to equol by human intestinal microflora—implications for health. *Molecular Nutrition and Food Research*.

[51] Shedd-Wise KM, Alekel DL, Hofmann H (2011). The soy isoflavones for reducing bone loss study: 3-Yr effects on pQCT bone mineral density and strength measures in postmenopausal women. *Journal of Clinical Densitometry*.

[52] Yamaguchi M, Lai YL, Uchiyama S, Nakagawa T (2008). Oral administration of phytocomponent p-hydroxycinnamic acid prevents bone loss in ovariectomized rats. *Molecular and Cellular Biochemistry*.

[53] Folwarczna J, Pytlik M, Zych M (2012). Effects of caffeic and chlorogenic acids on bone mechanical properties in female rats. *Bone*.

[54] Manolagas SC (2010). From estrogen-centric to aging and oxidative stress: a revised perspective of the pathogenesis of osteoporosis. *Endocrine Reviews*.

[55] Coxam V (2007). Current data with inulin-type fructans and calcium, targeting bone health in adults. *The Journal of Nutrition*.

[56] Takahara S, Morohashi T, Sano T, Ohta A, Yamada S, Sasa R (2000). Fructooligosaccharide consumption enhances femoral bone volume and mineral concentrations in rats. *The Journal of Nutrition*.

[57] Pelletier S, Tremblay GF, Bélanger G (2010). Forage nonstructural carbohydrates and nutritive value as affected by time of cutting and species. *Agronomy Journal*.

[58] Branham WS, Dial SL, Moland CL (2002). Phytoestrogens and mycoestrogens bind to the rat uterine estrogen receptor. *The Journal of Nutrition*.

[59] Burdette JE, Liu J, Lantvit D (2002). *Trifolium pratense* (red clover) exhibits estrogenic effects in vivo in ovariectomized Sprague-Dawley rats. *The Journal of Nutrition*.

[60] Shor D, Sathyapalan T, Atkin SL, Thatcher NJ (2012). Does equol production determine soy endocrine effects?. *European Journal of Nutrition*.

[61] Gu L, House SE, Prior RL (2006). Metabolic phenotype of isoflavones differ among female rats, pigs, monkeys, and women. *The Journal of Nutrition*.

[62] Leclercq G, de Cremoux P, This P, Jacquot Y (2011). Lack of sufficient information on the specificity and selectivity of commercial phytoestrogens preparations for therapeutic purposes. *Maturitas*.

[63] Wuttke W, Jarry H, Seidlová-Wuttke D (2007). Isoflavones-Safe food additives or dangerous drugs?. *Ageing Research Reviews*.

